# Quantifying the potential benefits of early detection for pancreatic cancer through a counterfactual simulation modeling analysis

**DOI:** 10.1038/s41598-023-46751-3

**Published:** 2023-11-16

**Authors:** Jiheum Park, Francesca Lim, Matthew Prest, Jennifer S. Ferris, Zainab Aziz, Alice Agyekum, Sophie Wagner, Roman Gulati, Chin Hur

**Affiliations:** 1 Department of Medicine, Columbia University Irving Medical Center, New York, NY 10032 USA; 2 Division of Public Health Sciences, Fred Hutchinson Cancer Center, Seattle, WA 98109 USA

**Keywords:** Cancer, Mathematics and computing, Medical research

## Abstract

The benefits of cancer early detection depend on various factors, including cancer type, screening method performance, stage at diagnosis, and subsequent treatment. Although numerous studies have evaluated the effectiveness of screening interventions for identifying cancer at earlier stages, there is no quantitative analysis that studies the optimal early detection time interval that results in the greatest mortality benefit; such data could serve as a target and benchmark for cancer early detection strategies. In this study, we focus on pancreatic ductal adenocarcinoma (PDAC), a cancer known for its lack of early symptoms. Consequently, it is most often detected at late stages when the 5-year survival rate is only 3%. We developed a PDAC population model that simulates an individual patient's age and stage at diagnosis, while replicating overall US cancer incidence and mortality rates. The model includes “cancer sojourn time,” serving as a proxy for the speed of cancer progression, with shorter times indicating rapid progression and longer times indicating slower progression. In our PDAC model, our hypothesis was that earlier cancer detection, potentially through a hypothetical screening intervention in the counterfactual analysis, would yield reduced mortality as compared to a no-screening group. We found that the benefits of early detection, such as increased life-years gained, are greater when the sojourn time is shorter, reaching their maximum when identification is made 4–6 years prior to clinical diagnosis (e.g., when a symptomatic diagnosis is made). However, when early detection occurs even earlier, for example 6–10 years prior to clinical diagnosis, the benefits significantly diminish for shorter sojourn time cancers, and level off for longer sojourn time cancers. Our study clarifies the potential benefits of PDAC early detection that explicitly incorporates individual patient heterogeneity in cancer progression and identifies quantitative benchmarks for future interventions.

## Introduction

Early detection of cancer can significantly improve survival by offering more effective treatment options. Screening has been the primary method for early detection, leading to a decline in mortality rates for multiple cancer types, such as cervical, colorectal, lung, and breast cancer since the introduction of their screening programs^1^.

Established screening programs are continuously investigated and evaluated to optimize outcomes in terms of effectiveness, efficiency, and safety. Randomized trials and cost-effectiveness analyses have been instrumental in evaluating the effectiveness of the screening strategies, considering factors such as target population, screening frequency, and screening initiation age^2–4^. Advances in medical technology, such as imaging technologies^5^, liquid biopsy^6, 7^, and artificial intelligence^8, 9^, further contribute to the development of more accurate, less invasive, or more cost-effective screening tests. However, there are no quantitative benchmarks available to directly answer specific counterfactual questions, such as: “If a patient diagnosed with stage IV cancer at age 60 had been diagnosed $x$ months/years earlier, what would their mortality benefits be?” Counterfactual analysis provides critical insight into the causal structure with which any intervention, such as screening programs, will interact^10^.

In this study, our objective was to provide a quantitative benchmark for early cancer detection by analyzing the aforementioned counterfactual questions in pancreatic ductal adenocarcinoma (PDAC). Recent research has shown promising strides in early detection, utilizing deep learning to analyze real-world longitudinal disease trajectory datasets^11^. These methods have demonstrated an impressive ability to predict diagnoses up to 3 years in advance. Our quantitative benchmark may provide guidance to inform a target timeline to optimize benefits of early detection for future PDAC interventions.

Unlike other prevalent cancer types in the U.S., such as colorectal, prostate, breast, and lung cancer, PDAC is characterized by an exceptionally high mortality rate, with a dismal 5-year survival rate of only 3–10%^12^. This is primarily due to the late-stage diagnosis and the aggressive nature of the malignant tumor. Ongoing improvements in survival rates for lung and breast cancers suggest that PDAC could become the second leading cause of cancer-related death by 2030^13^. In contrast to many other cancers, which have a wide array of treatment options including surgery, radiation therapy, chemotherapy, and immunotherapy, the only viable method for achieving long-term survival in PDAC patients currently is surgery in conjunction with perioperative chemotherapy^14^. Additionally, the natural progression of PDAC, including the duration of progression, remains unclear due to the absence of a well-established population screening program. This is in part because of its relatively low prevalence and the lack of an effective screening tool that can demonstrably reduce mortality from PDAC in the general population.

In accordance with our study objective, we developed a natural history model of PDAC that simulates US cancer incidence and mortality data, incorporating cancer sojourn time, with age and stage at diagnosis. Estimating cancer sojourn time, the duration between the onset of a detectable preclinical disease and its clinical diagnosis, is crucial for developing effective screening programs and optimizing early detection benefits^15^. However, direct observation of sojourn time is not feasible since disease onset is not observable.

To investigate the sojourn time for PDAC, most studies have primarily relied on modeling. For instance, Luebeck et al. employed the multistage-clonal-expansion (MSCE) mathematical model to illustrate tumor growth and progression dynamics, estimating a PDAC sojourn time of 3 years^16^. Another study estimated PDAC sojourn time by comparing the glycemic profiles of patients with and without cancer, assuming that the duration of hyperglycemia correlates with the time the tumor has been present. In this case–control study, researchers found that patients experienced hyperglycemia for an average of 30–36 months before PDAC diagnosis^17^. However, these estimates involve substantial uncertainty. For instance, in breast cancer, which has a well-established screening approach, reported sojourn time estimates vary widely, ranging from two to as many as 7.5 years^18, 19^. Given these uncertainties, we conducted a counterfactual analysis using three different mean sojourn times (3, 5, 10 years) incorporated in the natural history of PDAC. We then quantified the benefits of early detection at both the subgroup level (e.g., individual-level, age-groups) and the whole-group level (i.e., aggregated results).

## Results

### Modeling and simulation overview

**Figure 1:**
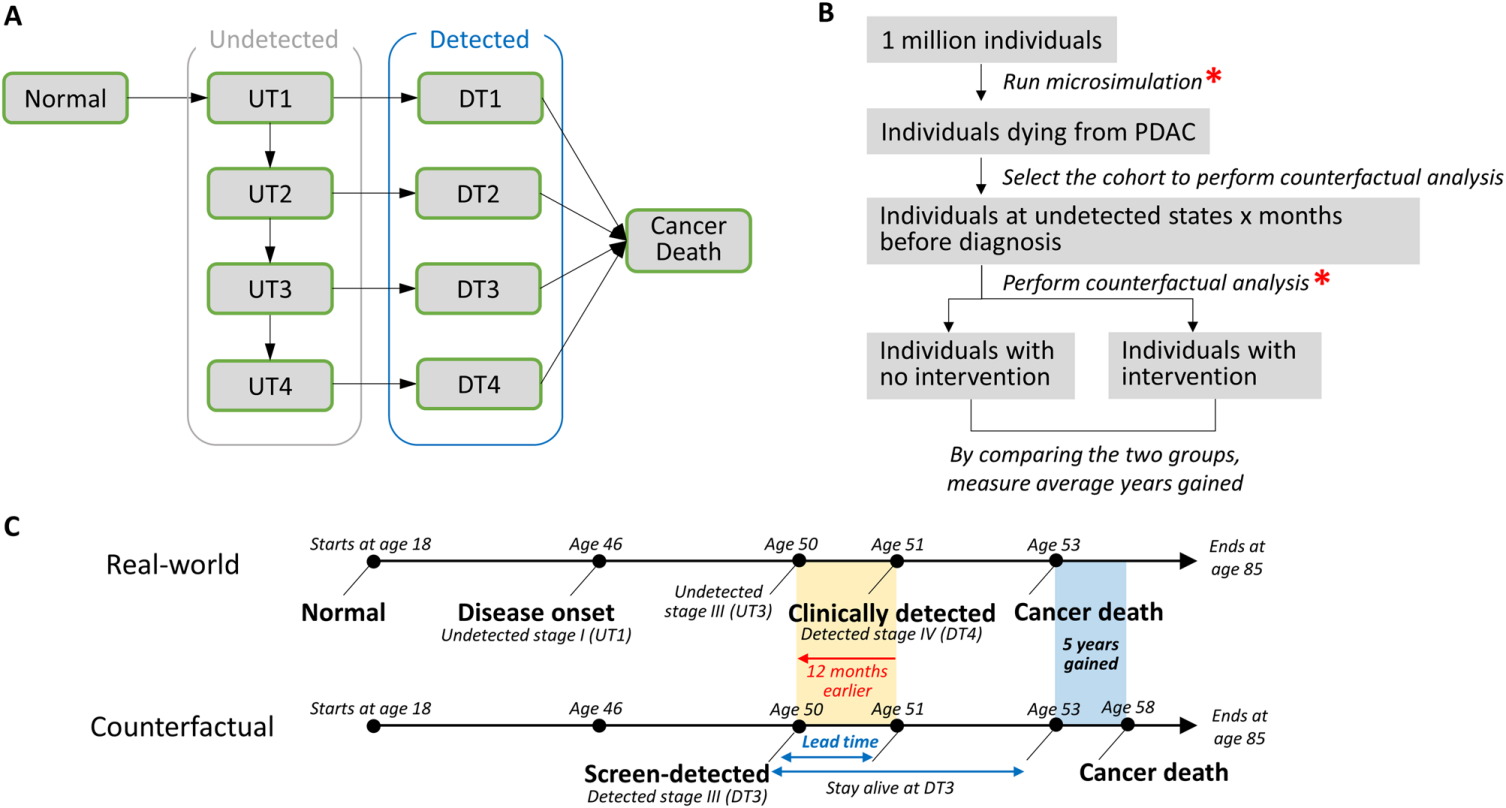
Study design for counterfactual analysis. (**A**) Natural history model for PDAC (Pancreatic Ductal Adenocarcinoma). The “undetected” states represent preclinical screen-detectable stages and “detected” states represent either clinically detected stages for example by symptoms in the real-world scenario or screen detected stages in the counterfactual world scenario. An arrow is used to indicate a possible transition from one state to another. *UT1* undetected stage T1, *UT2* undetected stage T2, *UT3* undetected stage T3, *UT4* undetected stage T4, *DT1* detected stage T1, *DT2* detected stage T2, *DT3* detected stage T3, *DT4* detected stage T4. (**B**) Counterfactual analysis using microsimulation, with a primary outcome of average years gained. The randomness (*) inherent in microsimulation and counterfactual analysis influences the sensitivity of the results. (**C**) Comparison of a real-world scenario with a hypothetical counterfactual world scenario. In the real-world scenario, a patient is clinically diagnosed with stage IV cancer at age 51. The counterfactual world scenario illustrates what could have happened if the same patient was detected 12 months earlier, potentially leading to an estimated additional 5 life-years.

We constructed a natural history model of PDAC, consisting of four preclinical, screen-detectable phases that progress to clinical staging and ultimately result in cancer death, as shown in Fig. 1A. We assumed that all preclinical stages were detectable through screening, and designed these stages to follow the defined cancer sojourn time. Meanwhile, the clinical stages were subjected to the age-dependent cause-specific mortality rate from the Surveillance, Epidemiology, and End Result (SEER), which accounts for treatments and therapies available during the data collection timeframe. Based on this design, we hypothesized that earlier cancer detection, possibly through a hypothetical screening intervention, would reduce mortality when evaluated through counterfactual analysis.

The model's transition probabilities were determined either directly using SEER data (e.g., age-dependent cancer-specific mortality for each stage, Fig. S1A) or by calibrating the model to age-dependent SEER PDAC incidence by stages (Fig. S1B) and overall mortality rates (Fig. S1C) (see “Methods” for more details). We ran a microsimulation model for 1 million individuals and conducted a counterfactual analysis on the cohort of patients who died from PDAC. By comparing counterfactual analysis results to the corresponding real-world scenarios, our primary outcome was the average life-years gained (Fig. 1B), as illustrated in Fig. 1C. The real-world scenario refers to the actual observed patient course informed by the natural history of PDAC model, while the counterfactual scenario refers to a hypothetical situation representing what might have happened under interventions.

### Characteristics of natural history of PDAC

**Figure 2:**
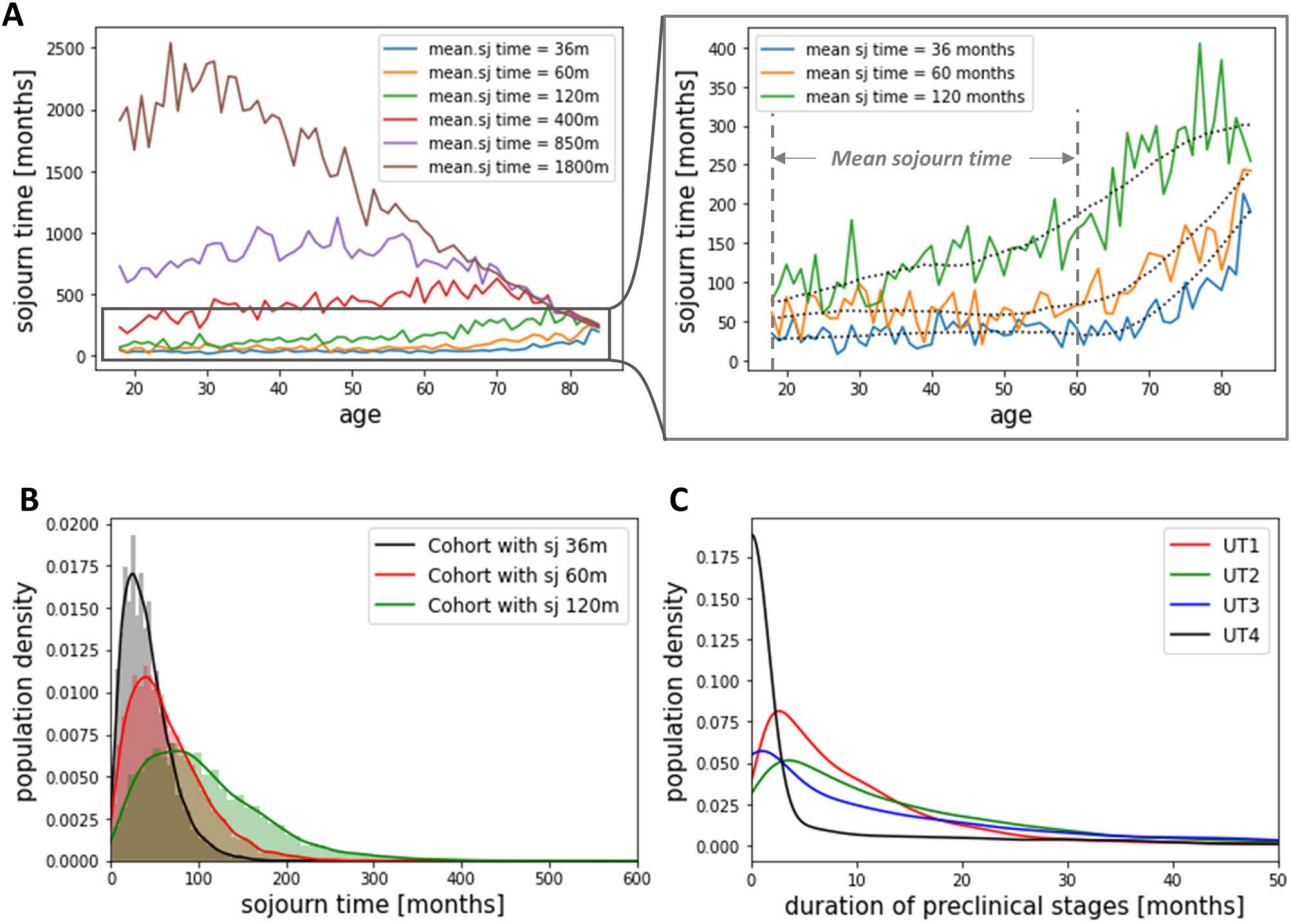
Characteristics of natural history of PDAC. (**A**) Calibration process of incorporating sojourn times in the natural history model of PDAC. Through our strategies of sequentially lowering the mean sojourn time, we generated multiple models with different mean sojourn times. We selected three models that represent PDAC natural history with mean sojourn times of 36 months, 60 months, and 120 months for our analysis. These models are referred to as sj36m, sj60m, and sj120m, respectively. The mean sojourn time is defined as the average value between ages 18 and 60, as there is a noticeable increase in sojourn time after age 60. We have depicted the age-dependent sojourn time trendlines for each model using dotted lines. (**B**) Sojourn time distributions for the selected cohort (i.e., individuals who died of pancreatic adenocarcinoma (PDAC)) from each model, used in the counterfactual analysis. The median values for each histogram are 36, 55, and 93 months. (**C**) Distribution of the duration of preclinical stages for the selected cohort from the sj36m model. The median duration of preclinical stage I, II, III, and IV are 7, 10, 7, 0 months respectively.

We estimated the sojourn times in the model using the mean first passage time (MFPT)^20^ in Markov chains, which is based on transition probabilities (see “Methods”). Through our systematic calibration process, we were able to sequentially lower the mean sojourn time to as low as 36 months while achieving a model fit to the target SEER data (Fig. 2A and Fig. S1). To investigate variations in early detection benefits across different sojourn times, we performed counterfactual analyses for three versions of the model with mean sojourn times of 36, 60, and 120 months, which we will refer to as sj36m, sj60m, sj120m, respectively. The resulting sojourn times, measured based on the MFPT in each model, displayed an increasing trend with age (Fig. 2A). As a simple verification, we evaluated the sojourn time distribution for the selected cohort (i.e., patients who died from PDAC) and found that the resulting median values were 36, 55, and 93 months, which are close to the targeted mean sojourn times (Fig. 2B). The histogram in Fig. 2B displays the proportion of patients with specific sojourn times, while the solid lines represent the density distribution for each sojourn time model. For example, within our 36-month sojourn time model (sj36m), the distribution of sojourn times spans from a brief 1 month to an extended 227 months, with a median of 36 months. Therefore, our model captures the variability and heterogeneity of sojourn times among different individuals. We also examined the duration of preclinical stages for the selected cohort, finding median durations less than a year for all preclinical stages (Fig. 2C).

### Counterfactual analysis on three models—sj36m, sj60m, sj120m

In all three model versions, approximately 10,000 people died from PDAC in a simulated population of 1 million patients for each model, representing a 1% lifetime risk of dying from the disease. This risk is marginally lower than the real-world statistic of 1.7%. This group of 10,000 patients constituted the cohort used in the counterfactual analysis for each model. However, to conduct a counterfactual analysis of patients' health states “x months earlier” than their original diagnosis (e.g., clinical diagnosis based on symptomatic presentation), these patients must be in one of the preclinical states at that specific time point. In the counterfactual scenario, we convert these preclinical “undetected” states at “x months earlier” to screen “detected” states (Fig. 1C) to assess the potential benefits of reduced mortality. Consequently, the number of eligible patients for the counterfactual analysis depends on each individual's health state at the time of the hypothetical screening intervention. For instance, if a patient's health state was “normal” 12 months before their clinical diagnosis, this patient must be excluded from the counterfactual analysis. Accordingly, as we turned back the clock from the time of clinical diagnosis (e.g., 3, 6, 12, …120 months before clinical diagnosis), the number of selected cohorts from each model for performing counterfactual analysis continually decreased sigmoidally (Fig. S2), as the probability of staying alive for an extended duration decreases. We observed a more significant drop of this number of selected cohorts in the model with shorter sojourn times (e.g., 8000, 6000, and 3000 are eligible for counterfactual analysis in sj120m, sj60m, and sj30m respectively at 12 months prior to clinical diagnosis, see Fig. S2). The smaller sample size led to wider confidence intervals in the analysis results.

### Whole-group analysis

#### Early detection benefits measured by life-years gained

**Figure 3:**
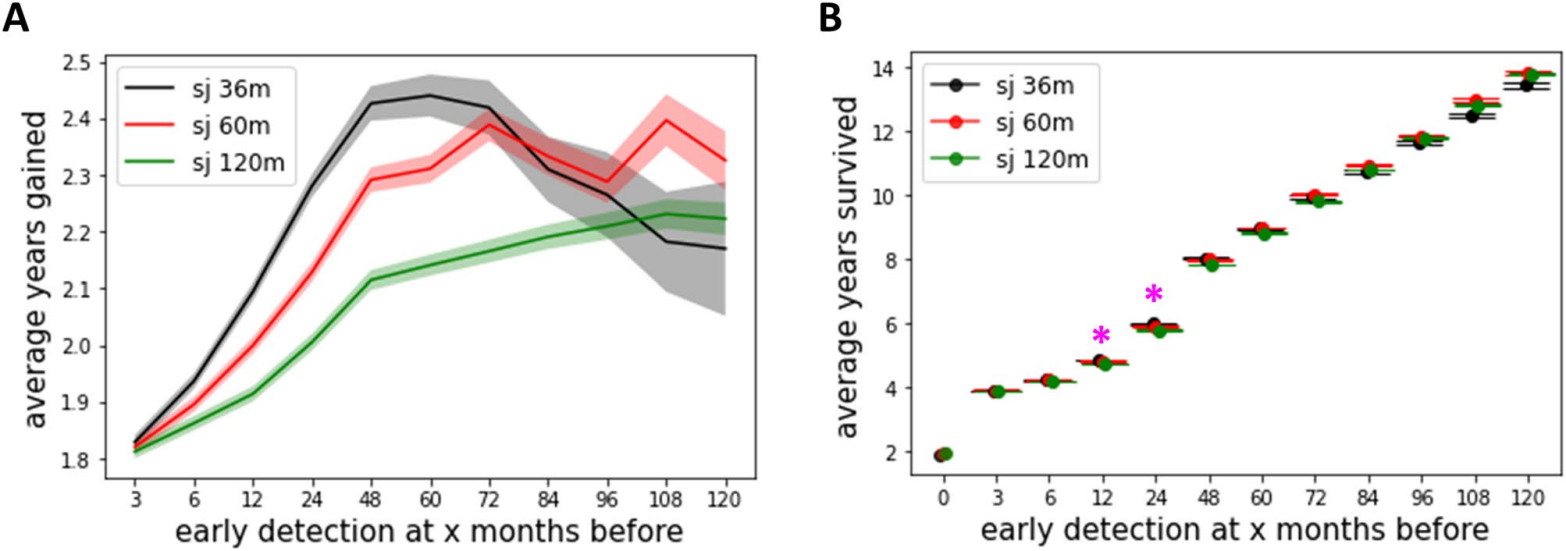
Simulation outcomes. (**A**) The benefit of early detection, estimated by average life-years gained for PDAC natural history model incorporated with mean sojourn times of 36 months, 60 months, and 120 months (i.e., sj36m, sj60m, sj120m). The average values are represented by solid line and confidence intervals are represented by shaded region. (**B**) The benefit of early detection, estimated by average survival length, which accounts for lead time bias. The sj36m model demonstrates significantly greater (p < 0.05) benefits at 12 and 24 months earlier interventions compared to the models with longer mean sojourn times, indicated by an asterisk (*).

The whole-group analysis demonstrated that early detection was most beneficial for cancer cases with shorter sojourn times (Fig. 3A). In the sj36m model, the maximum benefit (i.e., average life-years gained) was achieved when cancer was detected 4–6 years prior to the clinical diagnosis, resulting in an average of 2.4 life-years gained, which is significantly greater than those in the sj60m and sj120m models (i.e., 2.3 and 2.1 life-years gained, respectively) (Table 1). However, the maximum benefit from the sj36m model significantly decreased to below 2.1 life-years gained with earlier interventions, whereas the maximum benefit from the sj60m and sj120m models remained relatively stable.

**Table 1:** Simulation results: life-years gained from early detection.

Model	Screening intervention time prior to clinical diagnosis	Mean [years]	Median [years]	95% CI [years]
Sojourn time 36 months	6 months prior	1.94	1.25	1.92–1.95
	24 months prior	2.28	1.42	2.26–2.30
	60 months prior	2.44	1.58	2.40–2.48
Sojourn time 60 months	6 months prior	1.90	1.25	1.88–1.91
	24 months prior	2.13	1.33	2.11–2.14
	60 months prior	2.31	1.50	2.29–2.34
Sojourn time 120 months	6 months prior	1.86	1.25	1.85–1.87
	24 months prior	2.00	1.25	1.99–2.02
	60 months prior	2.14	1.33	2.12–2.16

#### Early detection benefits measured by survival duration

To examine the impact of lead time on early detection benefits, we measured survival duration, which incorporates lead time as the time from screening diagnosis to death (Fig. 1B). In contrast to the benefits measured by life-years gained, which displayed distinct patterns based on sojourn times, the benefits measured by survival duration exhibited a consistent sigmoidal pattern of increasing benefits across all models, regardless of their sojourn times. For example, when assessing benefits by survival duration, greater benefits with shorter sojourn times began at interventions 12 months before diagnosis and persisted only until 24 months before diagnosis. However, when measuring benefits by life-years gained, the model with shorter sojourn time showed significantly greater benefits (p < 0.05) compared to the model with longer sojourn time, starting from interventions at 3 months and continuing up to 60 months before diagnosis. Furthermore, the intriguing phenomenon observed with life-years gained—where maximum benefits were reached at intervention 4–6 years prior to diagnosis, then declined in the sj36m model while being maintained in the sj60m and sj120m models—was not seen when measuring early detection benefits by survival duration, as all three models displayed a consistent sigmoid-shaped pattern of increasing benefits.

### Subgroup analysis

#### Age-stratified analysis

**Figure 4:**
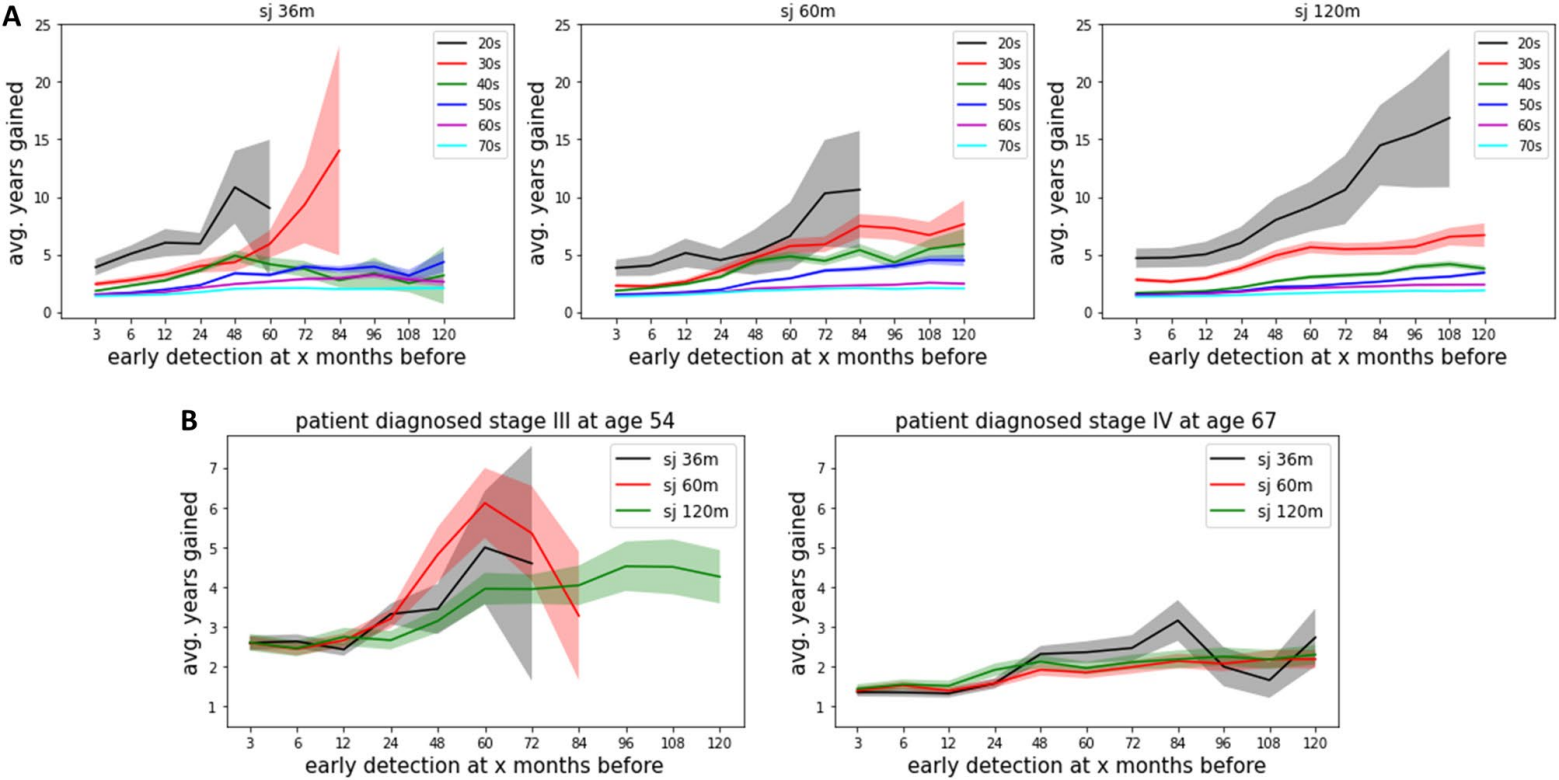
Early detection benefits quantified by subgroups. (**A**) Age-stratified analysis for 36-, 60-, and 120-month sojourn time models. (**B**) Individual-level analysis based on cancer stage and age at the time of diagnosis.

When we evaluated the average life-years gained by age group, we found that younger individuals experienced greater benefits (Fig. 4A). As compared to older age groups, younger age groups had increased uncertainty due to the relative rarity of PDAC in younger individuals.

#### Individual patient level-analysis

Based on simulation results from a population of 1 million patients, we can estimate the potential benefits of early cancer detection according to a patient's cancer stage and age at diagnosis. For example, our analysis suggests that if early detection is achieved, a 54-year-old patient diagnosed with stage III cancer is likely to gain more life-years than a 67-year-old patient diagnosed with stage IV cancer (Fig. 4B). However, predictions for earlier stages and younger patients may be less certain due to smaller sample sizes.

## Discussion

We present quantitative measures of the potential benefits of PDAC early detection using a counterfactual analysis. This analysis employed a microsimulation model that incorporated various sojourn times, calibrated to robust PDAC incidence, mortality, and survival data from the National Cancer Institute SEER database. Our systematic calibration approach demonstrated its capacity to determine model age-dependent transition probability sets that adequately reproduced SEER data, while representing a broad range of mean sojourn times, enabling the investigation of early detection benefits with highly variable sojourn timeframes. We examined the results at both a whole-group-level and subgroup-level to comprehensively analyze how sojourn time impacts PDAC early detection benefits.

Although the sojourn time for PDAC is thought to be short^16^, it can vary significantly among individuals with the same cancer type due to factors such as age, genetics, lifestyle, and biological differences in immune system responses^21^. Our model effectively addresses this individual variability, as demonstrated in Fig. 2B. The distribution of sojourn times in our example cohort for the 36-month sojourn time model (sj36m) ranges from as low as 1 month to a maximum of 227 months (Fig. 2B, black histogram), with the median value at 36 months. A 1-month sojourn time occurs when the patient is clinically detected at stage I (DT1) after residing in the undetected stage I (UT1) state for 1 month, which should be very rare. Indeed, this scenario accounts for only 0.2% of our simulation results.

In the counterfactual analysis, we discerned clear patterns of early detection benefits, which appeared to be contingent on the mean sojourn time of PDAC. For our three sojourn time models (sj36m, sj60m, sj120m), the early detection benefits increased until an intervention at 4–6 years prior to diagnosis. However, these maximum benefits significantly dropped (p < 0.05) afterward in the sj36m model, while they were relatively maintained in the longer sojourn time models (sj60m and sj120m models, Fig. 3A). We found that this decline of benefits in the sj36m model after the maximum benefit was reached, was due to the characteristics of the selected cohort. For instance, we could only perform counterfactual analysis with $x$ months earlier interventions on the cohort who had more than $x$ months of ‘undetected’ period (Fig. S2). The median age of cancer death for the cohort in sj36m model shifted to older ages as the intervention occurred earlier, while it remained relatively constant for the cohort in sj60m and sj120m models (Fig. S3). Specifically, the median age of cancer death for all three models at interventions close to the diagnosis (e.g., intervention at 3-months prior to diagnosis), was in the early 70s. However, in the cohort tested further in advance (e.g., intervention at 120 months prior to diagnosis), the median age of cancer death in sj36 model was nearly 80 years old, while those in sj60m and sj120m models were in the early and mid-70s, respectively. As age increases, the probability of benefiting from the intervention and gaining additional life-years decreases.

Our analysis also emphasizes the significance of addressing lead time bias in cancer screening and early detection studies. Lead time refers to the interval between disease detection through screening and the point at which it would have been clinically diagnosed, such as via symptoms, as illustrated in Fig. 1C. We assessed early detection benefits by calculating life-years gained, which does not include lead time. However, in practice, early detection benefits are often measured by survival duration from the point of diagnosis, which may incorporate lead time. This is because determining lead time is challenging, as the symptomatic presentation of a disease can vary significantly among individuals. With lead time involved, patients may appear to live longer when, in reality, their lives have not been extended, leading to an overestimation of a screening program's efficacy. This phenomenon, known as lead time bias, must be considered when evaluating the impact of screening programs on survival^22^.

Indeed, when we assessed early detection benefits using survival duration instead of life-years gained, the patterns observed based on life-years gained were no longer apparent. While early detection benefits quantified by life-years gained displayed notable differences in trends across sojourn time models along the various intervention months (Fig. 3A), those quantified by survival duration exhibited a consistent sigmoid-shaped pattern of increasing benefits across all three models (Fig. 3B). These discrepancies highlight the influence of lead time when evaluating screening programs. As such, our analysis can serve as a benchmark for assessing the extent of lead time bias involved in evaluating potential PDAC screening programs in the future.

While model-based approaches can enhance our understanding of complex problems like tumor dynamics, they may oversimplify the issues under numerous assumptions, as it is challenging to include all aspects of the problem. The mathematical modeling of PDAC natural history can span from the cellular level (e.g., cell growth, division, and death) to the population level (e.g., cancer epidemiology, including incidence, prevalence, and mortality rates)^23^; however, the natural disease course of PDAC remains elusive. While our model is more streamlined compared to the multifaceted PDAC model presented by Koopmann et al., which incorporates preinvasive, preclinical, and clinical stages under various model assumptions^24^, it accurately reflects the best available data. Despite our primary focus on replicating SEER data, our model naturally generates the mean duration of preclinical stages in a Weibull distribution shape (Fig. 2C), consistent with one of the assumptions incorporated in Koopmann et al.'s PDAC model. Further, the sojourn times obtained through calibration to SEER data exhibit an increasing trend with age (Fig. 2A), aligning with literature suggesting slower cancer progression at later ages^25, 26^.

Our analysis is also limited by its reliance on observed data. We designed clinical stages in our PDAC model to be subjected to the age-dependent cause-specific mortality rate from SEER, aiming to evaluate the mortality benefits of earlier detection. The cause-specific mortality rates from SEER adjust for the access to and impact of modern treatments available during the data collection period. Therefore, we assumed that earlier intervention would produce mortality benefits in counterfactual scenarios. However, it is important to note that SEER data is not updated in real-time, resulting in a potential lag between the availability of new treatments and their reflection in the mortality rates. Additionally, SEER data may not capture every nuance related to treatment access, such as regional differences in healthcare systems, insurance coverage, or other social determinants of health that could impact access to modern treatments.

The lack of validation using external data sources is another limitation. However, this is largely because most available real-world data on PDAC screening focuses on high-risk patients, as seen in studies such as the Cancer of Pancreas Screening-5 (CAPS5) study^27^. In contrast, our simulation model explores hypothetical screening in the general population, following the natural progression of PDAC. Therefore, directly comparing our results with existing real-world data is challenging due to the discrepancy in target populations. To provide some context, our model detected approximately 30% of cases at stage I. Meanwhile, the CAPS5 study—which focused on individuals with familial or genetic risks—reported 78% of detections at stage I. This underscores the potential for a significant increase in early-stage detections when screening targets high-risk populations. Additionally, the CAPS studies showed a median overall survival of 9.8 years for screen-detected PDAC patients. Our model reflected this survival rate when detection happened 5 years before clinical diagnosis—this timeframe yielded the most substantial benefits in our findings.

We did not perform a comprehensive cost-effectiveness analysis in this study, as there are currently no screening recommendations for PDAC. Our primary objective was to provide a quantitative benchmark for early detection using counterfactual analysis. A complete cost-effectiveness analysis would need to consider potential harms related to early diagnosis, such as quality detriments, complications that could lead to death or morbidity, and estimations of medical resource utilization and health costs associated with early detection efforts^28^. In our current study, the simulation model assumes perfect sensitivity for screening. This choice was made to explore specific “what if” scenarios, such as estimating the life years that could have been gained if a PDAC patient diagnosed at a later stage had been detected earlier. While our current model focuses on this aspect, it lays a strong foundation for future cost-effectiveness analyses. In subsequent studies, we intend to assess a variety of screening strategies with different sensitivities and specificities, evaluate treatment approaches tailored to cancer stages (e.g., based on tumor sizes), and consider the variability in sojourn times.

In summary, we developed a microsimulation model that incorporated age-varying sojourn time in the natural history of PDAC. In this model, we performed counterfactual analyses to provide a benchmark for early detection, measured in life-years gained. Our data can serve as a valuable tool for assessing the overall effectiveness of screening programs and modalities in the early detection of PDAC. Additionally, our methods have the potential to be applicable to other types of cancer and possibly other chronic diseases with preclinical states that could benefit from early detection.

## Materials and methods

### Study design

We constructed a patient-level microsimulation model with state transitions to perform counterfactual analyses on a cohort of patients who died from PDAC within the model. Every individual started in the normal health state with an initial age of 18 and was followed until age 85 or death. We simulated 1 million individuals in total. During each monthly cycle, individuals either remained in the same health state or transitioned to a new health state based on predetermined probabilities derived from published data sources or our model’s calibration process. The model consisted of four preclinical screen-detectable stages (UT1, UT2, UT3, and UT4 in Fig. 1A), which progressed to clinically detected stages (DT1, DT2, DT3, and DT4 in Fig. 1A), ultimately leading to cancer death, as illustrated in Fig. 1A. The possible state transitions in the model are Normal to UT1, UT1 to UT2, UT2 to UT3, UT3 to UT4, UT1 to DT1, UT2 to DT2, UT3 to DT3, and UT4 to DT4, as shown in Fig. 1A. Transitions to cancer death could only occur from clinically detected stages (DT1, DT2, DT3, and DT4).

### Model calibration and transition probabilities

We calibrated the model and estimated transition probabilities for each age bracket using population-based Markov model. We determined the sojourn time by utilizing these transition probabilities, leveraging the Markov chain mean first passage time (MFPT)^20^ methodology. We followed the MFPT formula shown in Eq. (1).

1 $${m}_{ij}=1+{\sum }_{k\ne j}^{s}{p}_{ik}{m}_{kj},$$

where ${m}_{ij}$ is average number of steps it takes to end up at state j given that current state is i.

We applied the transition probabilities, determined through the population-based Markov model, in our microsimulation approach. Individual transitions between states are determined based on these probabilities but also incorporate a degree of randomness, ensuring our model does not rigidly adhere to fixed sojourn times. Instead, our approach reflects a probabilistic representation of the sojourn time.

For the transition probabilities from the detected stages (i.e., DT1, DT2, DT3, and DT4 in Fig. 1A) to cancer death and all-cause mortality, we directly obtained estimates from the cause-specific survival data for PDAC provided by SEER^29^ and the 2017 U.S. life tables from Centers for Disease Control and Prevention (CDC)^30^ respectively. For the remaining transition probabilities, we obtained estimates by calibrating model outcomes of PDAC incidence by stages and overall mortality to SEER data, while sequentially lowering the mean sojourn times. The sojourn time in our model is defined as the time it takes for individuals to transition from undetected states to detected states for each cancer stage.

Due to the large parameter space of possible age-specific transition probabilities, the model lacks identifiability when using SEER data alone as a calibration target. Identifiability and model validity can both be improved with the addition of sojourn time estimates from literature.

According to the literature, the sojourn time for PDAC is estimated to be about 3 years. When we calibrated the model using data from SEER (overall mortality and incidence by stages) without including sojourn time as a calibration target, the transition probabilities resulting from this model give an estimate of the sojourn time that was about 1800 months based on the MFPT formula (Fig. 2A), which is much longer than the literature estimate for PDAC. While this model did align with SEER data, the lengthy sojourn time was biologically implausible, stemming from very low transition probabilities. This underscores the necessity of grounding our model with realistic sojourn time estimates from the literature to both enhance identifiability and ensure valid representations.

To reach sojourn time about 3 years, we needed sequential process of lowering the sojourn time. Our strategies to calibrate the model to the multiple calibration targets are detailed in 5 steps as shown below. We note that age-dependent PDAC mortality rates by stages were directly used as fixed transition probabilities from each detected stage (e.g., DT1, DT2, DT3, and DT4) to cancer death, which means these transition probabilities do not change throughout the calibration process.

- Step 1. Preparation of target data: We extracted SEER incidence data by stages for individual ages, which inherently contains noise. To mitigate the risk of overfitting due to noise, we generated trendlines for each stage, providing a more robust target dataset for our analysis. SEER data on the incidence of unknown PDAC, which accounts for considerable amount, was incorporated into the model by distributing it to stage I, II, III, and IV cases proportionally to their incidence at each age.
- Step 2. Age-invariant calibration: We performed a stepwise simulated annealing process using age-invariant transition probabilities (i.e., generating single values of transition probabilities that do not vary by ages) to refine our approximations before performing age-variant calibration in step 3. In step 2, we excluded sojourn time as a calibration target.
- Step 3. Age-variant calibration: Starting with the transition probabilities obtained from step 2, we performed a stepwise simulated annealing process using age-variant transition probabilities (i.e., generating transition probabilities that vary by age) until a satisfactory fit was visually confirmed as demonstrated in Fig. S1. In step 3, we excluded sojourn time as a calibration target.
- Step 4. Age-variant calibration for 100 iterations: We included the mean sojourn time as a calibration target in addition to SEER incidence and mortality and performed a stepwise simulated annealing process using age-variant transition probabilities for 100 iterations.
- Step 5. Sequential calibration process: We repeated step 3 and step 4 alternatively until we achieved the target mean sojourn time of 36 months.

As a result, we generated multiple models with different mean sojourn times (Fig. 2A), and selected three models representing PDAC natural history with mean sojourn times of 36 months, 60 months, and 120 months for our analysis. These models are referred to as sj36m, sj60m, and sj120m, respectively.

### Counterfactual simulations

To determine if an earlier diagnosis of PDAC leads to mortality benefits (e.g., life-years gained), we performed a counterfactual analysis using our microsimulation models (sj36m, sj60m, and sj120m). Comparing results from a counterfactual scenario to those in the real world, we measured average life-years gained and proportion of patients involved in lead time bias (i.e., cases with no life-years gained) at each month of screening intervention as primary outcomes (Fig. 1C). The real-world scenario refers to the actual situation following the natural history of PDAC model, while the counterfactual scenario refers to a hypothetical situation representing what might have happened under early interventions as illustrated in Fig. 1C. We tested screening interventions at 3, 6, 12, 24, 48, 60, 72, 84, 96, 108, and 120 months earlier.

We simulated 1 million individuals starting at age 18 in a normal health state and tracked their monthly health transitions until age 85 or death. We then identified individuals who died from PDAC in our simulation and recorded their age and month of diagnosis. To perform a counterfactual analysis of “what if we detected the patient’s PDAC earlier”, we tracked back each individual’s health state history $x$ months before their clinical diagnosis (e.g., symptomatic diagnosis). If the health state $x$ months before diagnosis was at one of the undetected states, we changed it to the corresponding detected state, but if it was normal, we excluded the patient from the counterfactual analysis. Thus, we selected a cohort eligible for counterfactual analysis for each month of screening interventions.

One specific example of implementing counterfactual analysis is that if a patient was diagnosed with detected stage IV (DT4) in our model, and 12 months before this diagnosis, the patient was in undetected stage III (UT3), we changed this state from UT3 to detected stage III (DT3). This meant the patient was now screen detected that month. We then continued the simulation until age 85, ensuring that patients remained alive in the model until the time they would have died from PDAC in the real-world scenario. This approach excluded rare instances where patients might die earlier in the counterfactual scenario than in the real-world, either due to all-cause mortality (e.g., heart attack, car accident, etc.) or from cancer, potentially resulting from the negative effects on their overall well-being caused by knowing about their cancer diagnosis earlier.

### Life-years gained vs survival duration

Using the counterfactual results, we assessed early detection benefits by measuring life-years gained and survival duration. Life-years gained represents the additional years of life an individual experiences in the counterfactual world compared to the real-world. Survival duration measures the time from the screening intervention to death in the counterfactual world. Individuals can die either from PDAC or all-cause mortality in the counterfactual world.

### Whole-group vs subgroup analysis

For each month of screening interventions, we selected a cohort eligible for counterfactual analysis and calculated life-years gained for each individual by comparing their outcomes in the counterfactual scenario to those in the real world.

For the whole-group analysis, we presented the average life-years gained for the selected cohort for each month of screening interventions in each model (sj36m, sj60m, and sj120m). For the subgroup analysis, we collected results for individuals belonging to a specific stage and age or age groups of interest and evaluated their average life-years gained. Since our model was run on a monthly cycle, patient age is represented as years and months. For example, if we want to assess the potential benefits of early detection for a patient who was diagnosed with PDAC stage III at age 53, we collect results for individuals diagnosed with PDAC stage III at ages ranging from 53 years and 0 months to 53 years and 11 months.

### Model sensitivity

There are two parts where inherent randomness of microsimulation models are involved in the analytical process, which we marked with red asterisks in Fig. 1B. Due to a relatively low incidence rate of PDAC, a microsimulation model with an insufficient number of individuals results in a greater degree of uncertainty in the results. We therefore simulated 1 million individuals, which resulted in successful alignment with the target data (Fig. S1). For the counterfactual analysis, we performed 10 iterations for each case and presented the mean values with 95% confidence intervals.

### Statistics

We performed an independent sample *t*-test for comparing the means of two groups and one-way ANOVA for comparing the means of three groups at the significance level of 0.05.

### Supplementary Information

Supplementary Information.

## Data Availability

All data, code, and materials used in the analysis are available at https://github.com/jp4147/counterfactual-analysis.
