# Supplementary material for: Quantifying the potential benefits of early detection for pancreatic cancer through a counterfactual simulation modeling analysis
Source: Sci Rep. 2023 Nov 16;13:20028. doi: 10.1038/s41598-023-46751-3 (PMC10654404; doi:10.1038/s41598-023-46751-3)
Supplement: Supplementary file 1 — Supplementary Information. [file 41598_2023_46751_MOESM1_ESM.docx]

Supplementary Material


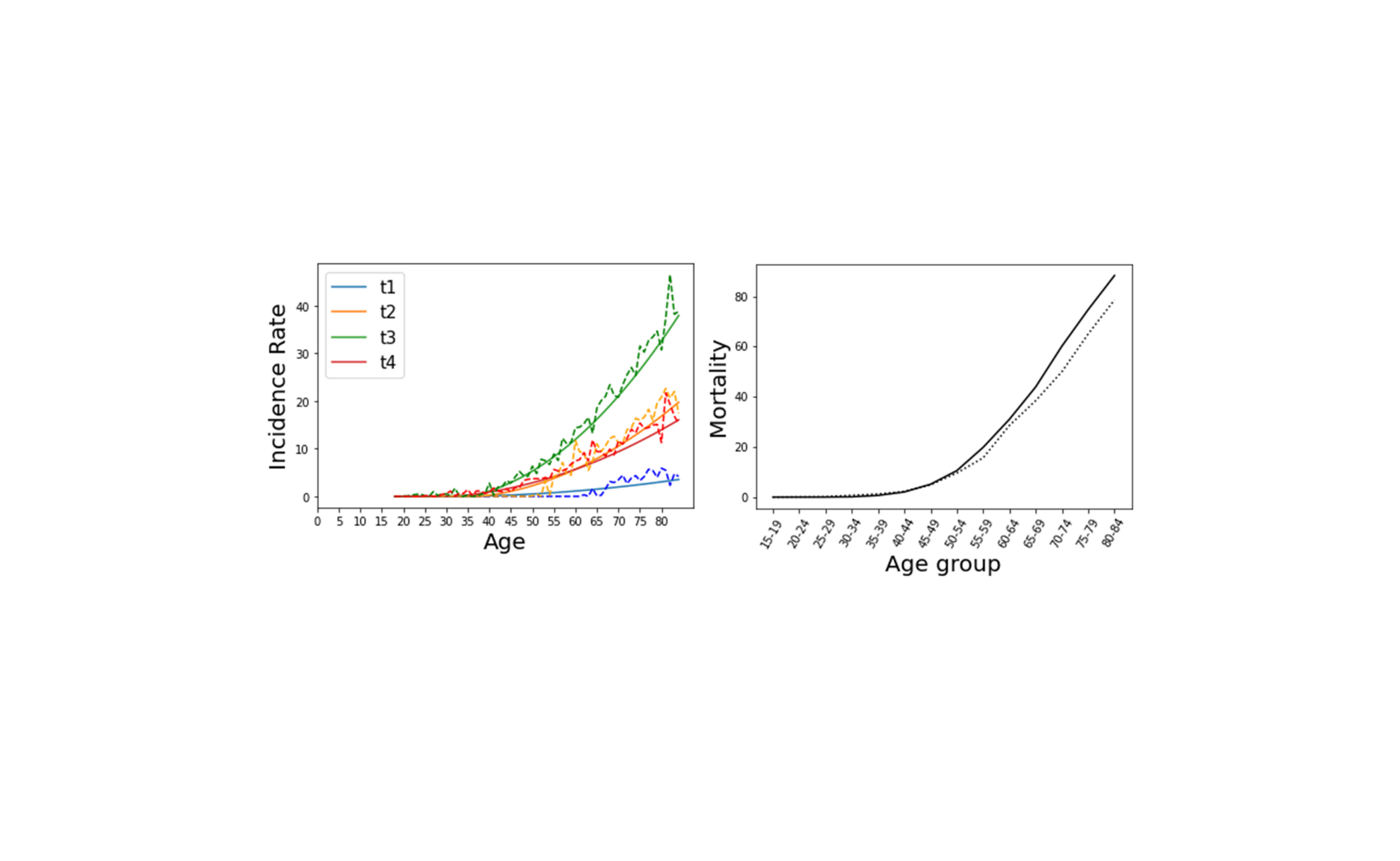

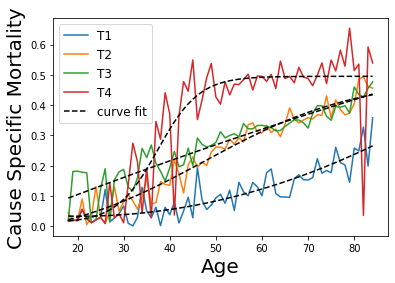


C

B

**A**

Fig S1. The Surveillance, Epidemiology, and End Result (SEER) data used in the model. (A) Age-dependent cancer-specific mortality for each stage. We used the values from fitted line as transition probabilities from “detected” states to cancer death (Fig 1A). (B) Incidence by stages. The solid line represents the target data from SEER, while the dotted line shows the simulated results. (C) Overall mortality by age groups. The solid line represents the target data from SEER, while the dotted line shows the simulated results.


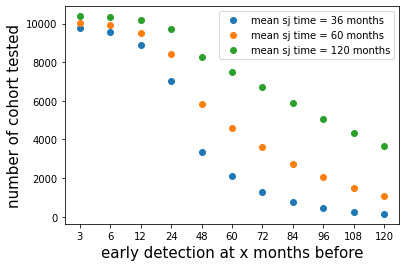


Fig S2. Number of selected cohorts eligible for counterfactual analysis using 36-month, 60-month, and 120-month sojourn time models at each month of earlier interventions.


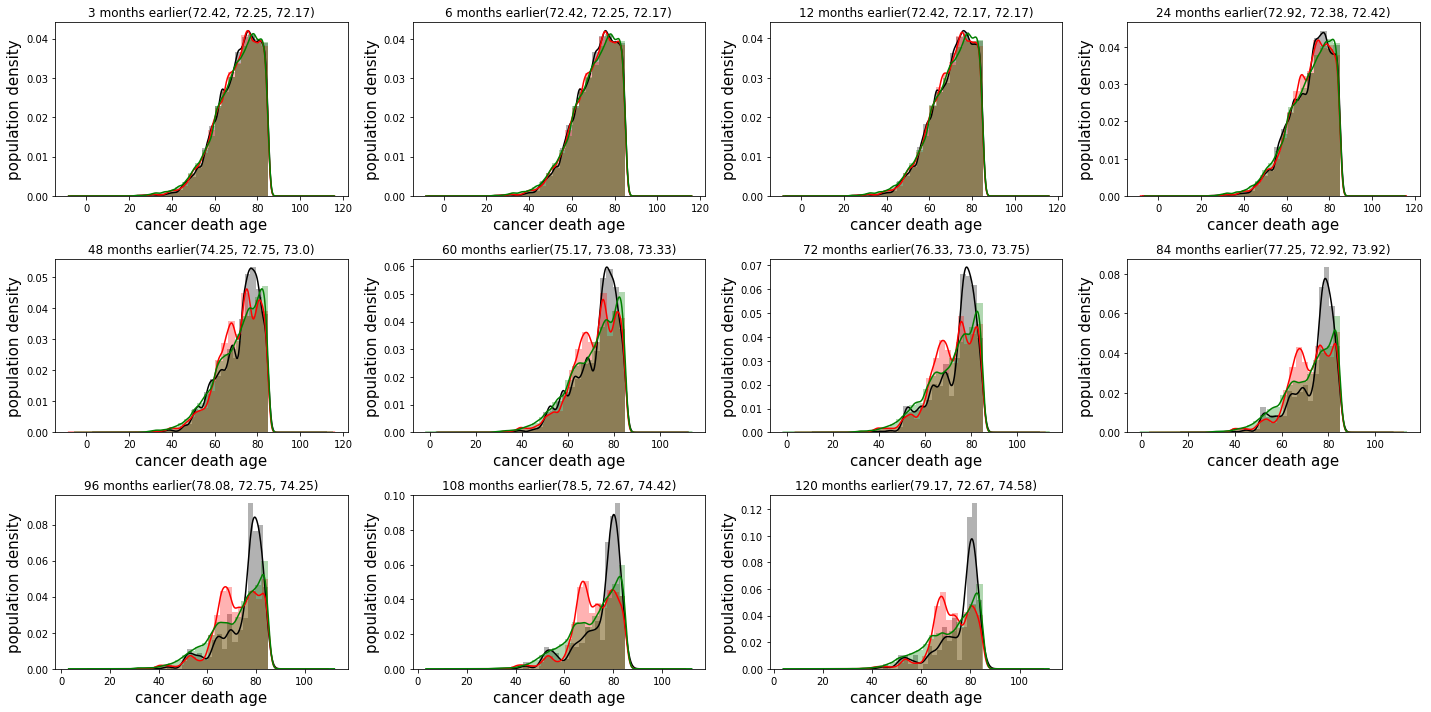


Fig S3. The distribution of cancer death age for a cohort from the sojourn time 36-month model (black), 60-month model (red), and 120-month model (green). The median values of each histogram are listed in the order of the 36-, 60-, and 120-month models.

Table S1. Calibrated transition probabilities

|  | Mean | Median | 95% CI |
| --- | --- | --- | --- |
| Normal to UT1 | 0.000250 | 0.000007 | 0.000003-0.000014 |
| UT1 to UT2 | 0.136001 | 0.090931 | 0.055000-0.125543 |
| UT2 to UT3 | 0.086461 | 0.062271 | 0.049898-0.076146 |
| UT3 to UT4 | 0.039370 | 0.007323 | 0.001915-0.007924 |
| UT1 to DT1 | 0.000286 | 0.000004 | 0.000004-0.000017 |
| UT2 to DT2 | 0.004649 | 0.000195 | 0.000111-0.000544 |
| UT3 to DT3 | 0.035982 | 0.024767 | 0.009883-0.022518 |
| UT4 to DT4 | 0.067694 | 0.043575 | 0.033659-0.057858 |

Due to the skewed nature of the data, we derived 95% confidence intervals (CI) using a log-normal distribution. This table excludes mortality transition probabilities as they were not calibrated. Instead, they were determined directly using either SEER data or 2017 U.S. life tables from CDC.
